# Sparsity-Based Pixel Super Resolution for Lens-Free Digital In-line Holography

**DOI:** 10.1038/srep24681

**Published:** 2016-04-21

**Authors:** Jun Song, Christine Leon Swisher, Hyungsoon Im, Sangmoo Jeong, Divya Pathania, Yoshiko Iwamoto, Misha Pivovarov, Ralph Weissleder, Hakho Lee

**Affiliations:** 1 Center for Systems Biology, Massachusetts General Hospital, Boston, Massachusetts, United States of America; 2 School of Engineering and Applied Sciences, Harvard University, Cambridge, Massachusetts, United States of America; 3 Department of Radiology, Massachusetts General Hospital, Boston, Massachusetts, United States of America; 4 Department of Systems Biology, Harvard Medical School, Boston, Massachusetts, United States of America

## Abstract

Lens-free digital in-line holography (LDIH) is a promising technology for portable, wide field-of-view imaging. Its resolution, however, is limited by the inherent pixel size of an imaging device. Here we present a new computational approach to achieve sub-pixel resolution for LDIH. The developed method is a sparsity-based reconstruction with the capability to handle the non-linear nature of LDIH. We systematically characterized the algorithm through simulation and LDIH imaging studies. The method achieved the spatial resolution down to one-third of the pixel size, while requiring only single-frame imaging without any hardware modifications. This new approach can be used as a general framework to enhance the resolution in nonlinear holographic systems.

Lens-free digital in-line holography (LDIH) is an emerging technology for point-of-care medical diagnosis^1^,^2^,^3^,^4^. The technology records holograms of objects directly placed on imagers, and digitally reconstructs the images of the objects. With no intermediate optical components between objects and imagers, LDIH is simple, compact, cost-effective, and capable of large field-of-view imaging. Recent applications of the LDIH technique has shown its potential for biomedical applications by molecularly profiling cancer cells^3^ and morphologically discerning a wide range of organisms (*e.g.*, bacteria, blood cells, *C. elegans*, plankton)^1^,^5^,^6^.

The spatial resolution of LDIH is limited by the physical size of individual sensing elements (pixels) on a semiconductor imaging sensor (an imager). Spatial information smaller than a pixel is lost in the imaging process, because the recorded holograms are sub-sampled versions of the true hologram. While imagers with smaller pixels could improve resolution, the approach is challenged by practical issues such as prohibitive device cost, heating from high density pixels, and often reduced field-of-view. Alternatively, new methods for resolution enhancement have been explored. For example, a sequence of low resolution images were numerically reconstructed^7^,^8^,^9^; the pixel function of an imaging sensor was predetermined and a deconvolution was used to improve spatial resolution^10^. Although these methods achieve sub-pixel resolution, they require additional hardware components, and increase the complexity of image acquisition.

Compressed sensing (CS) is a new signal recovery method^11^,^12^ that has been applied to super-resolution imaging on a wide range of platforms. Based on the sparse nature of images, CS can improve spatial resolution from under-sampled measurements^11^,^12^. This approach is particularly appealing for hologram reconstruction, because it entails no modification to the optical setup and reduces data acquisition time. CS has been applied to conventional holographic systems, including off-axis holography, multiple view projection holography, and Fresnel holography^5^,^13^,^14^,^15^,^16^. Directly applying CS algorithms to LDIH, however, is a challenging task. LDIH is inherently a nonlinear system; unlike conventional holography, which preserves both the intensity and the phase of light, LDIH records the light intensity only. Conversely, most CS algorithms require linearity and random sampling to accurately reconstruct data, and thereby are incompatible with LDIH^17^,^18^.

Here, we report on a new computational framework for super-resolution on LDIH systems. This approach uses the sparse nature of images to recover high frequency components. Specifically, we devised an algorithm compatible with non-linear LDIH by adopting the *L*_0_-norm minimization. The developed algorithm achieved higher resolution (up to 300%) than conventional reconstruction methods, recovering both amplitude and phase information at sub-pixel resolution. We validated the approach through numerical simulation, and applied it to images acquired with a LDIH system. The algorithm required only a single image taken with uniform sampling, and could be readily combined with existing LDIH systems without any hardware modification. Such features could facilitate the point-of-care application of LDIH by enabling simpler optical setup and faster image acquisition. Furthermore, this method can be used as a general framework to enhance resolution in non-linear holographic systems.

## Results

### System setup

**Figure 1:**
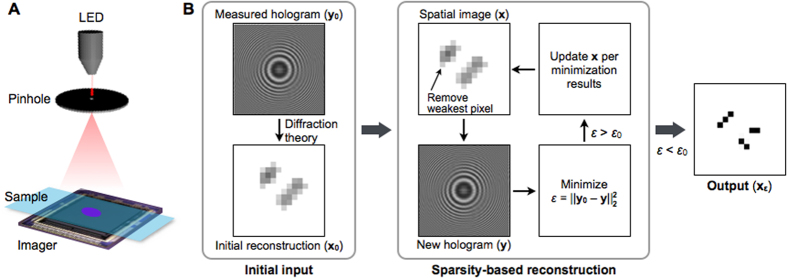
Sparsity-based algorithm to enhance the spatial resolution in lens-free digital inline holography (LDIH). (**A**) Imaging schematic of a typical LDIH system. The system consists of a light-emitting diode (LED), a pinhole, and an imager. A sample is placed directly above the imager, and the hologram coming from the sample is recorded. (**B**) Block diagram of the developed algorithm. The measured hologram (**y**_**0**_) and its numerical reconstruction (**x**_**0**_) via diffraction theory are used as an initial input. The sparsity-based routine executes an iterative minimization. In each iteration, the algorithm first increases the sparsity of the spatial image (**x**) by removing the weakest pixel and generates a new hologram (**y**). The algorithm then minimizes the difference (*ε*) between **y** and **y**_**0**_ by adjusting the pixel values of the spatial image at a given sparsity. The combination of pixel values that produces smallest *ε* is selected to update **x**. The iteration stops when *ε* becomes smaller than the threshold value *ε*_0_, and the final output (**x**_**ε**_) is obtained.

The LDIH imaging system (Fig. 1A) has simple optics, consisting of a light-emitting diode (LED), a pinhole, and a semiconductor imaging sensor. The pinhole is used to generate a coherent spherical wavefront. The interference between the incident light and the scattered light from the sample is the observed hologram^19^. The imager is positioned directly underneath the sample to record the hologram, and the object image is numerically reconstructed (see Methods for details). Without a lens between the sample and the imager, the system has a unit magnification to offer a wide field-of-view^3^. The imaging resolution, however, is limited by the discreteness of imager pixels; features smaller than the pixel size are under-sampled and only blurred images can be reconstructed.

### Sparsity algorithm for LDIH

We reasoned that the *L*_0_-norm minimization, which is compatible with non-linear systems, could be adapted to improve the spatial resolution from LDIH. The *L*_0_-norm minimization requires a sparse input signal to reconstruct the missing information. As an input, we thus chose the object image which is generally sparser than the holographic pattern. The relationship between the observed hologram (**y**) and the object image (**x**) was modeled as **y** = |**B·H·x**|, where **H** denoted the hologram operator. **B** is a low-pass, or blur, filter that accounts for the down-sampling effect coming from the discrete size of the imager pixels. We then solved the following optimization problem:


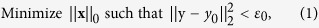


where ||**x**||_0_ is the nonzero-counting *L*_0_-norm of **x**, namely the number of nonzero elements in vector **x**, which also represents the sparsity of the signal; ||**y** − **y**_**0**_||_2_^2^ (=*ε*) is the difference between the measured (**y**_**0**_) and the estimated (**y**) holograms; and *ε*_0_ is the error threshold. This optimization aims at finding the sparsest possible solution of **x** (i.e., reconstructed image) but penalizes estimations that deviate significantly from the measured hologram by imposing *ε < ε*_0_.

To numerically solve Eq. (1), we developed a two-constraint iterative scheme (Fig. 1B), which finds the optimal minimization of the *L*_0_-norm (||**x**||_0_) while maintaining data consistency by minimizing *L*_2_-norm (||**y** − **y**_**0**_||_2_^2^) at each step. As an initial input, the algorithm uses an object image (**x**_**0**_) reconstructed from a measured hologram (**y**_**0**_) via diffraction theory (see Methods for details). It then executes an iterative process. The first step, thresholding, removes a pixel in the object image (**x**), whose value is closest to the background. This reduces the number of non-zero elements in **x** and thereby results in a smaller ||**x**||_0_ (*L*_0_-norm minimization). The second step, shrinking, finds the optimal **x** that minimizes *ε* (the change in the hologram), while keeping ||**x**||_0_ constant (*L*_2_-norm minimization). The found **x**, which is sparser than the initial input, is then used as a new input. The algorithm exits the iteration when *ε* is smaller than the predetermined threshold (*ε*_0_), yielding the final object image (**x**_**ε**_).

### Algorithm optimization

To handle the large number of pixels within an image, we adopted the Limited-memory Broyden–Fletcher–Goldfarb–Shannon (*L*-BFGS) method, which can solve large scale nonlinear minimization problems^20^. *L*-BFGS is a quasi-Newton algorithm designed for optimization problems with large number of variables. We applied *L*-BFGS to find **x** that minimizes *ε* (*=*||**y** − **y**_0_||_2_^2^). Note that *L*-BFGS enforces data consistency between the measured (**y**_**0**_) and the newly estimated (**y**) holograms; sparsity is maintained through the *L*_0_-norm constraint. We also implemented a fast-computing algorithm based on TwIST (Two-step Iterative Shrinking and Thresholding) to improve computational speed for wide field-of-view images^21^.

We found that a key criterion for stable computation was to establish an optimal exit condition (*ε < ε*_0_) from the iterative loop. As a quantitive measure of precision (convergence), we defined the difference (*ε*) from a pixel-by-pixel comparison:


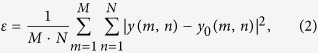


where *y*(*m*, *n*) and *y*_0_(*m*, *n*) are the components of **y** and **y**_**0**_, respectively, at the spatial coordinate index (*m*, *n*). *M* (and *N*) is the total number of rows (and columns) in the hologram matrix. We then set the the threshold (*ε*_0_) such that the average precision for all pixels was >98%; with this condition, all test images converged to their final solution.

**Figure 2:**
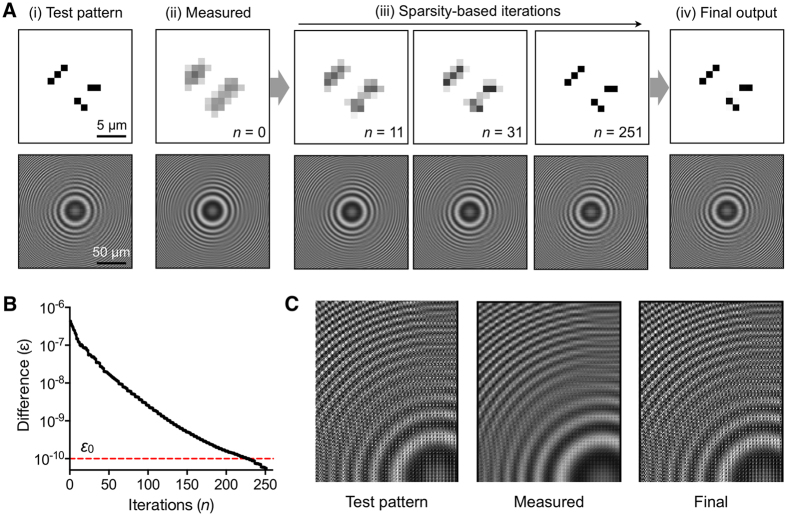
Sparsity-based reconstruction. (**A**) [i] The true object image and its hologram without signal loss. The smallest feature size in the object is 1.2 μm. [ii] A measured hologram is simulated for a 2.4-μm pixel imager. The object image is then reconstructed from the diffraction theory. These images are used as an input to the sparsity-based algorithm. [iii] The sparsity-based reconstruction progressively improves the image resolution. [iv] The object image is recovered after 251 iteration steps. (**B**) The difference (*ε*) between a new and the measured holograms decreased over the iteration steps. The threshold value (*ε*_0_) used here corresponds to <2% error for all pixels. (**C**) Zoom-ins of the test-pattern, the measured, and the final holograms. Starting from the measured hologram, the algorithm restored high frequency components.

Figure 2 shows an example of the iterative process using a simulated object. The test pattern (Fig. 2A-i) had the smallest feature size of 1.2 μm, and we calculated an ideal hologram (no-loss). We then simulated the LDIH measurement with the imager pixel size of 2.4 μm (Fig. 2A-ii). This step produced a down-sampled hologram (**y**_**0**_) and its corresponding reconstructed image (**x**_**0**_). We next applied the sparsity algorithm using **x**_**0**_ and **y**_**0**_ as an input. With each iteration, the resolution of the spatial image progressively improved (Fig. 2A-iii). With the improvement of the spatial resolution, the difference (*ε*) between the simulated hologram (**y**) and the reconstructed hologram (**y**_**0**_) decreased (Fig. 2B). The algorithm finally recovered the original pattern, even though its inputs were blurred holograms (Fig. 2C). We also tested the algorithm in the presence of pixel noise. We added varying levels of Gaussian noise to all pixels in the hologram (**y**_**0**_). The algorithm could recover the original object even at the noise level of 5% in signal intensity (Fig. S1).

### Spatial Resolution

**Figure 3:**
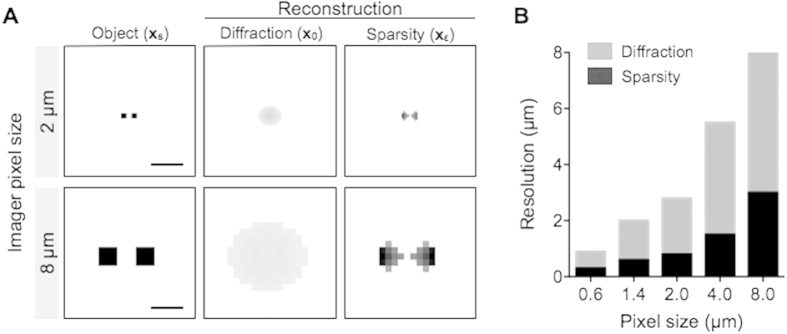
Resolution limit. (**A**) The true object images (**x**_**s**_, left column) contained two squares whose sizes and inter-distance were smaller than the imager pixel sizes (top row, 2 μm; bottom row 8 μm). Diffraction-based reconstruction (**x**_**0**_, middle column) could not resolve the original pattern. Applying the sparsity-algorithm to **x**_**0**_, two separate squares could be resolved (**x**_**ε**_, right column). Scale bar, 2 μm (top) and 8 μm (bottom). (**B**) Comparison of the spatial resolution with and without sparsity-based reconstruction. The pixel size was varied from 0.6 to 8 μm. Note that the sparsity-based algorithm achieves sub-pixel spatial resolution. The following simulation conditions were used: light wavelength, 405 nm; the distance between objects and the detection, 0.5 mm.

We next determined the resolution enhancement achievable by the sparsity algorithm. As a true object image (**x**_**s**_), we positioned two objects at a distance smaller than the pixel size (Fig. 3A, left), and then simulated a measured hologram (**y**_**0**_ = |**B**·**H**·**x**_**s**_|) according to the discrete pixel size of the LDIH. The initial reconstruction of the object image (**x**_**0**_) through the Rayleigh diffraction (see Materials and Methods) could not resolve the objects (Fig. 3A, middle). The resolution of the LDIH is primarily determined by the physical pixel size, even though the holograms were up-sampled via linear interpolation. Subsequent application of the sparsity algorithm, however, could separate these objects (Fig. 3A, right). To find the resolution limit, we varied the distance between the objects as well as the simulated pixel size. The sparsity algorithm showed a sub-pixel resolving power (Fig. 3B), giving two- to three-fold increase in resolution across a range of detector pixel size (0.6–8 μm).

### Simulation studies

**Figure 4:**
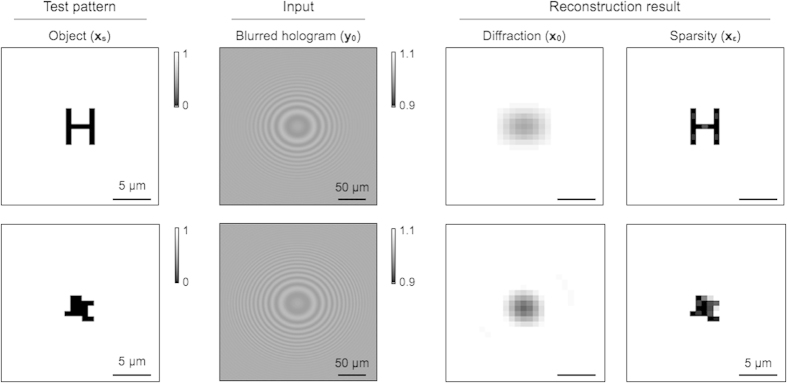
Reconstruction of sub-pixel patterns. Opaque patterns in a transparent background were used as object images (**x**_**s**_). The smallest feature size was 0.8 μm. Holograms were simulated for a 2.4-μm pixel imager. The reconstruction based on Rayleigh-diffraction only produced blurred images (**x**_**0**_), whereas the sparsity algorithm recovered sub-pixel features (**x**_**ε**_).

We validated the developed algorithm through numerical studies. We first simulated binary images consisting of transparent and opaque patterns. Large or irregular patterns with the smallest feature size of 0.8 μm were used. Holograms were calculated and processed with a low pass filter (**B**) to simulate LDIH measurements with a 2.4-μm pixel imager. Conventional reconstruction produced blurred object images, as the high frequency signal was lost in the simulated input holograms. The sparsity-based reconstruction, on the other hand, could reliably recover the lost high frequency information, consistently achieving three-fold resolution enhancement (Fig. 4).

We further extended the algorithm to recover the object phase as well as the magnitude. The phase information is particularly relevant in biological applications, because it can enhance contrast between different objects of similar sizes (*e.g.*, biological cells *vs.* labeling microspheres). To incorporate phase reconstruction, we used a complex object vector (**x**) whose components are


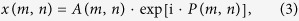


where *A*(*m*, *n*) and *P*(*m*, *n*) are components of the amplitude (**A**) and the phase (**P**) matrices, respectively, at the spatial position index (*m*, *n*). We modified the reconstruction algorithm to include both **A** and **P** matrices in its routine; the algorithm minimizes the number of nonzero elements both in **A** and **P**, and adjusts the pixel values in **A** and **P** to minimize *ε*. These matrices were not treated independently: **A** was used to determine the weakest pixel to remove, and the decision propagated to **P**.

**Figure 5:**
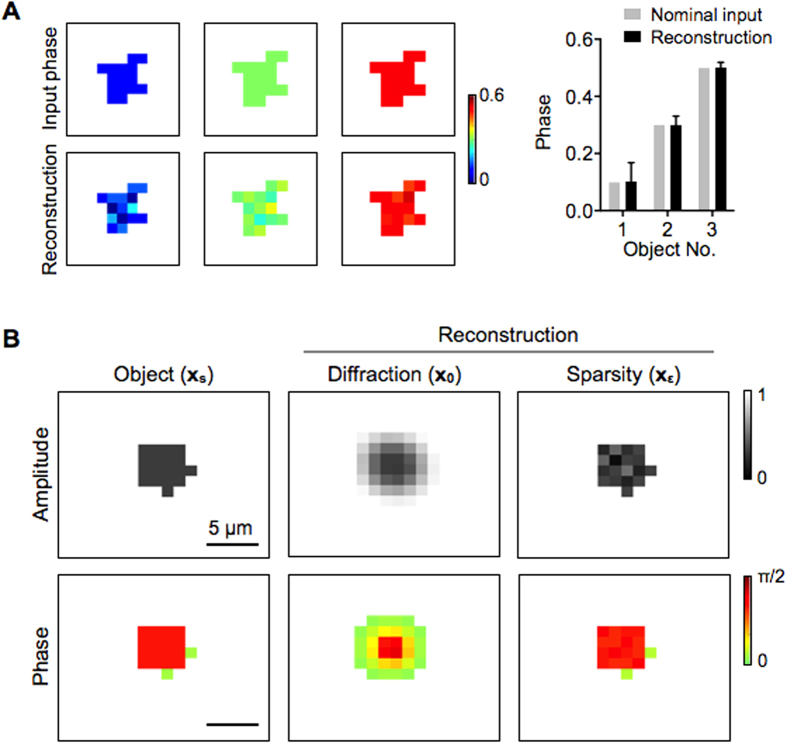
Reconstruction of sub-pixel patterns with phase. The sparsity algorithm was extended to recover both the amplitude and the phase of object patterns. (**A**) Objects with a uniform, nominal phase values (0.1, 0.3 and 0.5) were tested. From simulated holograms, the sparsity algorithm recovered the phase information. The relative error decreased, as the phase contrast between the object and the background increased. (**B**) A non-symmetric pattern with sub-pixel features and varying phases was tested. The smallest feature size was 1.1 μm. The algorithm not only achieved sub-pixel resolution, but also restored accurate amplitude and phase values. Holograms were simulated for a 2.2-μm pixel imager.

We tested the extended algorithm using simulated objects with homogeneous but different phase values (Fig. 5A, left). Holograms were calculated assuming an imager with 2.2-μm pixel size. The extended algorithm accurately recovered the phase information (Fig. 5A, right). Note that the relative error decreased as the phase differences between the object and the background increased. We further simulated an object with varying phase distributions with feature size (1.1 μm) smaller than the imager pixel size (Fig. 5B). The algorithm reconstructed both amplitude and phase, while maintaining the capacity for sub-pixel resolution enhancement. The computational load was approximately twice of the amplitude-only version as the number of variables doubled.

### Sub-pixel imaging with LDIH

**Figure 6:**
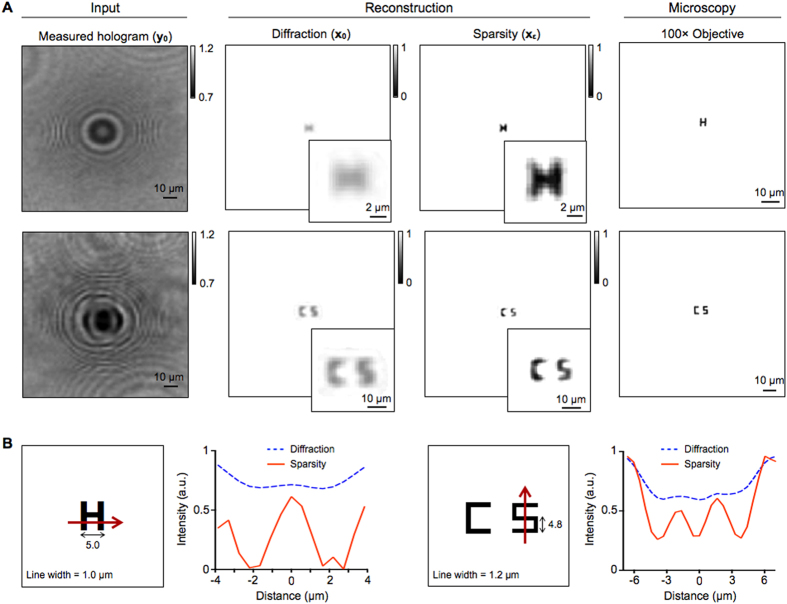
Reconstruction of sub-pixel patterns from LDIH images. (**A**) The sparsity algorithm was tested on images acquired with a LDIH system, which showed sub-pixel resolution enhancement. The reconstructed images were compared to reference images acquired by a bright field microscope with a 100x objective. (**B**) Schema of object and line profiles from LDIH show improved resolution after applying the sparsity algorithm.

We applied the sparsity algorithm to reconstruct actual LDIH images. We used a LDIH setup with a 2.2 μm pixel complementary metal-oxide-semiconductor (CMOS) imager to acquire holograms (see Materials and Methods). We first used a standard test pattern (USAF1951) to check the resolution enhancement. We acquired holograms of parallel line patterns, and compared reconstructed images (Fig. S2). The sparsity algorithm recovered all patterns that have varying widths and spacings. We next imaged complex patterns with sub-pixel line width. The objects were patterned on glass substrates via electron-beam lithography followed by metal deposition. Conventional, diffraction-based reconstruction (**x**_**0**_) failed to resolve winding geometries (*e.g.*, ‘H’, ’S’; Fig. 6A). The sparsity-based processing (**x**_**ε**_), however, restored the sub-pixel patterns, and achieved the resolution close to reference images acquired by a bright field microscope with a 100 × objective lens.

### High resolution imaging of biological objects

**Figure 7:**
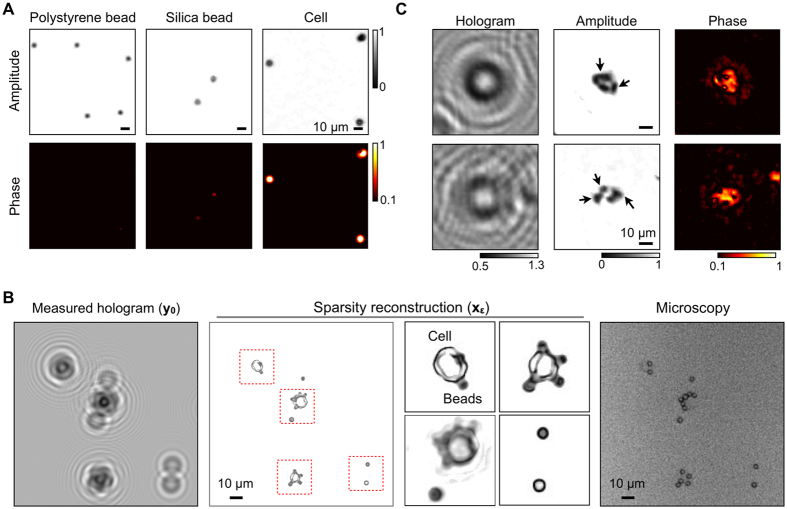
High resolution reconstruction of biological samples. (**A**) Objects with different optical transmittance and phase contrasts were imaged: polystyrene bead, silica bead, and a mammalian cell. The sparsity algorithm recovered the optical signature, allowing for the object identification. (**B**) Cancer cells were labeled with polystyrene beads, and imaged by a LDIH system (left). The sparsity algorithm recovered both the amplitude and the phase information of the sample (middle). Cells and beads could be differentiated from the size as well as the optical contrast (inset). The reconstructed image was comparable to the reference image acquired by a bright field microscope with a 100x objective. (**C**) The sparsity algorithm was applied to detect intracellular targets. Cell nucleus was stained with a chromophore to enhance the optical contrast. The reconstructed LDIH image shows high contrast between the cell nucleus and cytosol indicated by arrows.

We further applied the sparsity algorithm to objects with varying phases. Initial tests employed simple spherical objects: polystyrene microbeads (diameter, *d* = 4 μm), silica microbeads (*d* = 5 μm), and leukocytes (*d* = 10 μm). Following the hologram acquisition, we used the extended sparsity algorithm to recover both amplitude and phase information. The reconstructed images had high contrast, allowing us to distinguish these objects (Fig. 7A). Note that the large field-of-view (24 mm^2^) of LDIH, combined with high resolution reconstruction, enabled us to detect >10^4^ micrometer-scale objects in a single image acquisition (Fig. S3).

We next prepared mixed-phase objects by labeling cells with polystyrene microbeads conjugated with antibodies (see Materials and Methods). The labeled cells produced non-symmetric, complex diffraction patterns (Fig. 7B, left). In the reconstructed images (Fig. 7B, middle), however, cells and beads were clearly identified, which was further validated by bright field microscopy (100 ×; Fig. 7B, right). We could also differentiate the nucleus from cytoplasm in a cell (Fig. 7C). To enhance the optical contrast, we stained cell nuclei with a chromophore (see Materials and Methods). As the chromophore reduced the optical transmittance, cell nuclei appeared darker than cytoplasm in the reconstructed image.

## Discussion

We have developed a sparsity-based algorithm for LDIH resolution enhancement. This new method can improve the resolution of LDIH down to one-third of the imager pixel size, and recovers both the magnitude and the phase of objects. Importantly, the sparsity method provides sub-pixel resolution enhancement from a single-frame measurement, without requiring additional hardware or image acquisition steps. The algorithm empowers the LDIH system to achieve the imaging capacity close to high-resolution microscopy, and is effective for imaging sensors with a broad range of pixel size.

Sparsification of images generally has a sharpening effect, which, if unrestricted, could introduce artifacts. We minimize such risks by enforcing data consistency (||**y**_**0**_ − **y**||_2_^2^) between the measured (**y**_**0**_) and the estimated holograms (**y** = |**B** · **H** · **x**|) while fulfilling a sparsity constraint (||**x**||_0_). This approach is key to achieving high resolution that conforms to true object images. In the current application, we used a sparse object image (**x**) as an input. The developed algorithm, however, could also be applied to less-sparse images. In those cases, the sparsity requirement can be fulfilled with a basis (Φ) transformation, such that Φ**x** is sparse instead of **x** itself. Wavelet transformations, such as Daubechies Wavelets, are extensively used methods for such purposes^22^,^23^.

With the developed algorithm, LDIH could achieve a resolving power close to that of high magnification optical microscopy (100 ×) while retaining the benefit of a large field-of-view. This is a significant advance from our previous system whose resolution was comparable to that of 20 × microscopy^3^. The high resolution and the capacity to recover object’s optical property could lead to many biological applications. For example, LDIH could be a platform for multiplexed cellular analysis. We could simultaneously profile multiple surface markers by labeling cells with microbeads of different sizes and optical signatures. Furthermore,intracellular markers could be detected after staining with chromophores.

We anticipate that the present work will provide a framework for applying sparsity-based methods to nonlinear holographic systems. The developed method can be further improved by i) adopting other up-sampling processes (*e.g.*, floating pixel) to prepare an improved initial input (**x**_**0**_) to the sparsity algorithm^24^; ii) expanding the algorithm to process non-sparse samples through mathematical transformations (*e.g.*, wavelet transformation); and iii) porting the numerical routine to parallel computing (*e.g.*, graphic processing unit) to enable real-time image reconstruction. Such developments would significantly aid in the point-of-care applications of LDIH platforms, allowing for faster and more accurate diagnostics.

## Materials and Methods

### Construction of a LDIH system

The LDIH system consisted of a light-emitting diode (LED), a pinhole, and an imager. The illumination central wavelength of LED was 405 nm. A pinhole (aperture diameter, 100 μm) was placed in front of the LED to generate a coherent spherical wavefront. Samples were positioned 10 cm under the pinhole and 1 mm above an imager (Aptina Imaging MT9P031). The imager had 2592 × 1944 pixels with the pixel size of 2.2 × 2.2 μm^2^.

#### Preparation of test patterns

Samples with test patterns were created on glass substrate. Patterns were transferred to the slides via electron-beam lithography (Raith-150, Raith) followed by metal deposition (Ti, 10 nm; Au, 100 nm). The size of patterned features were measured via scanning electron microscopy (Ultra 55, Zeiss).

#### Cell labeling with microbeads

SkBr3 human cancer cells were labeled first with biotinylated anti-EpCAM antibody followed by streptavidin-coated polystyrene particles (7 μm diameter, Spherotech), each for 10 min at room temperature. Leukocytes were prepared from 0.6 mL blood samples mixed with 12 mL BD Phosflow Lyse/Fix buffer (1 ×) for 15 min at 37 °C. The cells were resuspended in PBS.

#### Cell staining

5 × 10^5^ lymphoma cells (Daudi) were placed on a glass slide. Adhered cells were fixed and permeabilized using Foxp3/Transcription Factor Staining Buffer Set (00-5523-00, Affymetrix) per manufacturer’s protocol. Subsequently, cells were labeled with anti-Ki67 antibody (556003, BD) at 4 °C, followed by 3 × wash and incubation with biotinylated anti-mouse secondary antibody (BA-2000, Vector Laboratories). Cells were washed again and incubated with ABC peroxidase (PK6100,Vector Laboratories). Finally, AEC + substrate-chromogen (K3464, Dako) was added for color development.

#### Diffraction-based reconstruction

The reconstruction routine that we previously developed for a smartphone-imaging system^3^ was adopted to the LDIH system. In brief, we recorded two holograms, one without samples (a reference image) and the other with samples (a target image). A normalized hologram was then obtained by calculating a pixel-by-pixel ratio between the target and the reference images. The normalized hologram was up-sampled four times through linear interpolation, and entered an iterative phase-retrieval routine^25^,^26^. First, the normalized hologram was numerically back-propagated for an optical distance between the object and the imager planes. The field propagation was based on the Rayleigh–Sommerfeld diffraction theory; the optical field was calculated by the inverse Fourier transform of the multiplication between the Fourier transform of field and the transfer function. Second, physical constraints (optical transmission 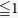) were applied to the back-propagated image to correct for artificial twin image. Third, the updated image was propagated to the imager plane to produce an updated hologram with nonzero phase information. The process was then repeated using the updated hologram as a new input. The iteration stopped when retrieved phase information converged. A typical iteration number was 10~30.

#### Sparsity-based reconstruction

We created a custom-designed package (in MATLAB) to perform the resolution enhancement. The measured hologram and the object image obtained through the conventional reconstruction (described above) were used as an input to the sparsity-based processing. The core of the package was the sparsity-based reconstruction, an iterative program seeking to find the optimized object image. The optimized object image was determined by two constraints: it needs to be sparsest possible solution which minimizes the error between the newly estimated hologram and the measured hologram. Through the iterations, the image sparsity was gradually decreased by merging the weakest pixel of each iteration into background. After each change in image sparsity, the program estimated a new hologram, and minimized the error value between estimated and measured holograms by adjusting the intensity of non-background pixels. The computation was run on a workstation with Intel^®^ Xeon^®^ EP processors and 64 GB memory (Lenovo ThinkStation S30).

## Additional Information

**How to cite this article**: Song, J. *et al.* Sparsity-Based Pixel Super Resolution for Lens-Free Digital In-line Holography. *Sci. Rep.* **6**, 24681; doi: 10.1038/srep24681 (2016).

## Supplementary Material

Supplementary Information
