# Supplementary material for: Sparsity-Based Pixel Super Resolution for Lens-Free Digital In-line Holography
Source: Sci Rep. 2016 Apr 21;6:24681. doi: 10.1038/srep24681 (PMC4838824; doi:10.1038/srep24681)
Supplement: Supplementary Information [file srep24681-s1.pdf]

SPARSITY-BASED PIXEL SUPER RESOLUTION FOR LENS-FREE DIGITAL IN-LINE HOLOGRAPHY

Jun Song[1,2¶], Christine Leon Swisher[1,3¶], Hyungsoon Im[1,3], Sangmoo Jeong[1,3], Divya Pathania[1,3], Yoshiko Iwamoto[1], Misha Pivovarov[1,3], Ralph Weissleder[1,3,4], Hakho Lee[1,3*]

[1] Center for Systems Biology, Massachusetts General Hospital, Boston, Massachusetts, United States of Amcerica

[2] School of Engineering and Applied Sciences, Harvard University, Cambridge, Massachusetts, United States of America.

[3] Department of Radiology, Massachusetts General Hospital, Boston, Massachusetts, United States of America

[4] Department of Systems Biology, Harvard Medical School, Boston, Massachusetts, United States of America

Corresponding author:
H. Lee, PhD
hlee@mgh.harvard.edu

¶ These authors contributed equally to this work.

SUPPLEMENTARY FIGURES

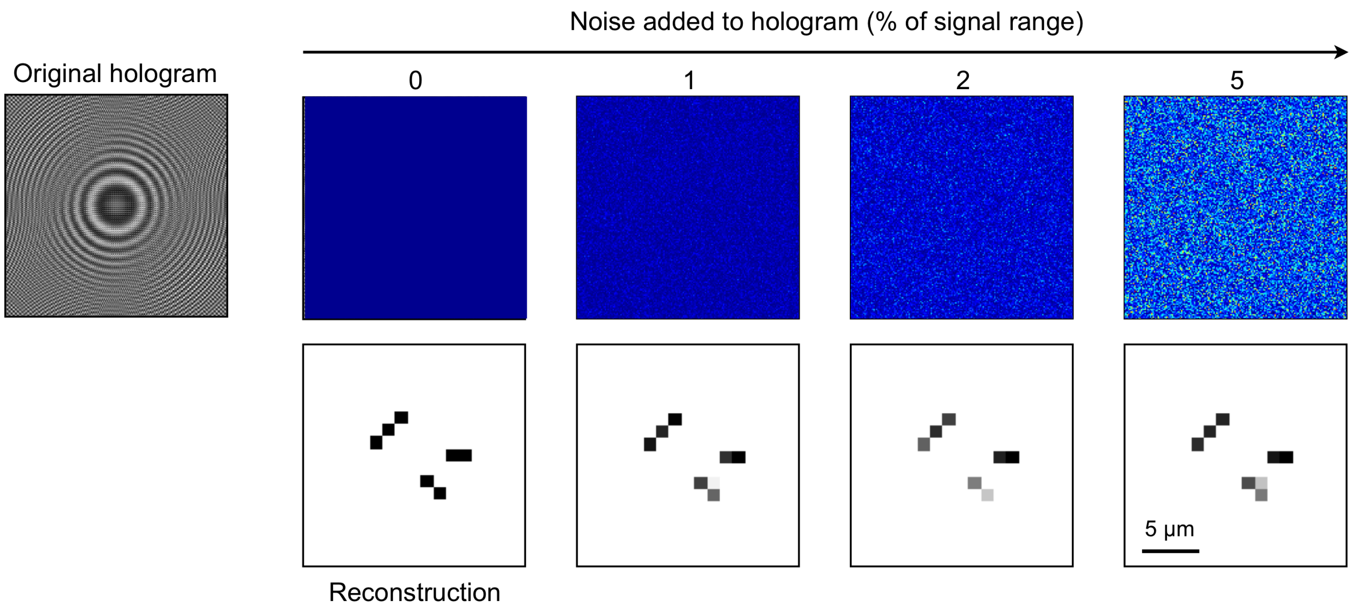

Fig. S1. Noise test of the sparsity algorithm. Gaussian noise was added to the original hologram input (left) before the sparsity-based reconstruction. The noise level was varied in proportional to the signal range in the hologram. In this example, the sparsity algorithm tolerated up to 5% of the noise level.

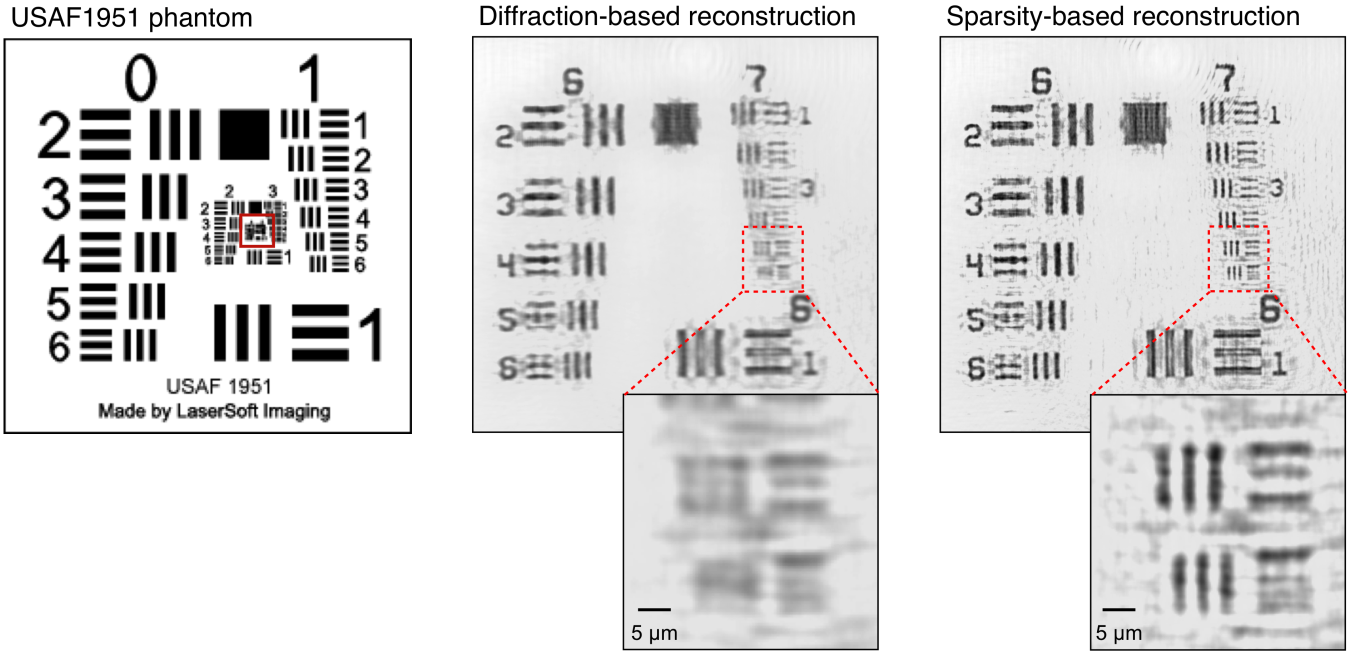

Fig. S2. Reconstruction of an USAF1951 phantom. The test patterns in the red solid box (left) were imaged by the LDIH system, and reconstructed by the diffraction-based (middle) and the sparsity-based (right) algorithms. The sparsity method showed higher resolving power than the diffraction method. The sparsity method resolved all patterns including the smallest feature with the line width of 2.1 μm.

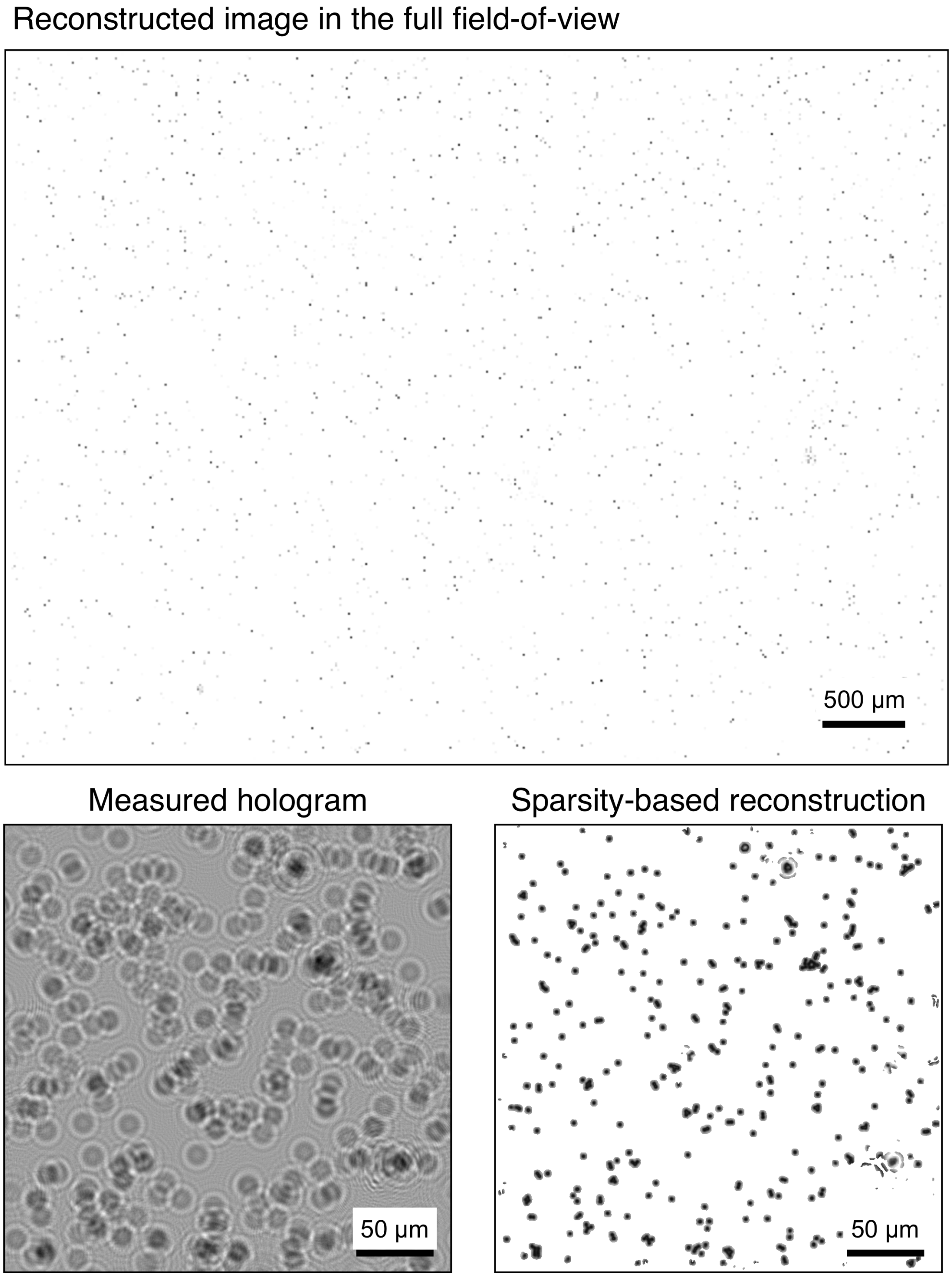

Fig. S3. Wide field-of-view imaging. With its unit magnification, the LDIH supports wide field-of-view imaging. We reconstructed >10^6 microbeads in a single view of 6 mm^2 (top). The sparsity algorithm was applied to reconstruct a high resolution object image (bottom).
